# Timed walk as primary outcome measure of treatment response in clinical trials for HTLV-1-associated myelopathy: a feasibility study

**DOI:** 10.1186/s40814-015-0031-1

**Published:** 2015-10-23

**Authors:** Fabiola Martin, Eisuke Inoue, Irene C. M. Cortese, Ramon de Almeida Kruschewsky, Adine Adonis, Maria Fernanda Rios Grassi, Bernardo Galvão-Castro, Steven Jacobson, Yoshihisa Yamano, Graham P. Taylor, Martin Bland

**Affiliations:** 1 Department of Biology, Centre for Immunology and Infection, Hull York Medical School, University of York, York, UK; 2 National Centre for Child Health and Development, National Medical Centre for Children and Mothers, Research Institute, Tokyo, Japan; 3 National Institute of Neurological Disorders and Stroke, National Institutes of Health, Bethesda, USA; 4 Advanced Laboratory of Public Health, Gonçalo Moniz Center, Fundação Oswaldo Cruz, Salvador, Bahia Brazil; 5 Bahian School of Medicine and Public Health (EBMSP), Salvador, Bahia Brazil; 6 Department of Rare Diseases Research, Institute of Medical Science, St. Marianna University Graduate School of Medicine, Kawasaki, Japan; 7 Department of Medicine, Section of Virology, Imperial College London, London, UK; 8 Department of Health Sciences, University of York, York, UK

**Keywords:** HTLV, Myelopathy, HAM/TSP, 10-metre walk test, Sample size, Clinical trial, Rare disease

## Abstract

**Background:**

To advance the treatment of HTLV-1-associated myelopathy/tropical spastic paraparesis (HAM/TSP), randomised controlled therapeutic studies with appropriate and sensitive outcomes are reuired. One candidate outcome is the 10-metre walk test (10MWT), a patient-centred, simple and functional measure. To calculate sample size based on 10MWT as the primary outcome, variability within and between subjects must be known.

**Methods:**

Data on 10MWT from 76 patients with HAM/TSP were prospectively collected from four specialist centres in Brazil, Japan, USA and UK. Data, collected at two time points, 6 months apart, were log transformed and subjected to analysis of covariance.

**Results:**

Baseline mean (standard deviation = SD), median 10MWT were 23.5 (18.9), 16.3 s/10 m and at 6 months 24.9 (23.9), 16.4 s/10 m. The mean (SD) % increase in walk time was 5.74 % (28.2 %). After logarithmic transformation, the linear correlation between baseline and 24 weeks 10MWT was *r* = 0.938. Using these data, it was determined that a randomised controlled trial with 30 participants per group would have 90 % power to detect a 19 % decrease or a 23 % increase in 10MWT.

**Conclusions:**

The intra-patient variability of 10MWT is relatively small in HAM/TSP over 6 months. 10MWT is a feasible outcome measure for a clinical trial in HAM/TSP. To our knowledge, this is the first ever recommendation for the sample size required for trials in HAM/TSP patients.

## Background

HTLV-1-associated myelopathy/tropical spastic paraparesis (HAM/TSP) is a neuroinflammatory disease of the spinal cord caused by the retrovirus human T lymphotropic virus type 1 (HTLV-1) [1]. HTLV-1 is also known to be the cause of an aggressive haematological malignancy, adult T cell leukaemia/lymphoma (ATLL) [2]. Having some features similar to multiple sclerosis, and often being of insidious onset, HAM/TSP is difficult to diagnose. Symptoms are usually confined to the lower body. Patients typically complain of stiffness and weakness of the legs, falls, pain in the lower back and legs, urinary urgency, constipation and erectile dysfunction. The majority of patients develop a chronically progressing disability, and 50 % become wheelchair-dependent within 2 decades of onset. A small subset progresses rapidly and a third group minimally [3]. Specific diagnostic criteria have been developed to differentiate HAM/TSP from other myelopathies [4].

Thirty years after the discovery of HTLV, there is still no internationally recognised treatment available for HTLV-1 infection or HAM/TSP. Clinical scientists are keen to find effective therapy for HAM/TSP. This is ideally proven through a double-blind, randomised, controlled trial (DB RCT) with a well-defined, primary outcome measure, tested in a sufficiently large, well-described, patient cohort. Designing such a trial for HAM/TSP patients has been proven challenging, as addressed by Martin et al. previously [5].

In the process of designing a HAM/TSP clinical trial that meets all the desirable criteria, an international HAM/TSP clinical trial study group (HAM/TSP CTSG) consisting of HAM/TSP experts from Brazil, Japan, UK and USA was formed. The aim of this group was to collaboratively design a DB RCT approved by all consortium members and to gain access to a large enough HAM/TSP patient cohort. This would allow adjustment of outcome of HAM/TSP patients by variables such as subgroup, gender and baseline disability as well as a short trial recruitment phase, to minimise trial costs.

There is no uniformly recognised reliable biomarker of treatment response in HAM/TSP. The HAM/TSP CTSG therefore chose to evaluate the potential of a clinical outcome measure, the 10-metre walk test (10MWT) as a primary outcome measure. 10MWT measures the time a patient takes to walk 10 m in a straight line using their usual walking aid and is presented as seconds/10 m. A decrease in 10MWT thus represents improved mobility. 10MWT was chosen for three reasons: (i) When surveyed HAM/TSP patients felt that improving their gait should be a primary goal of a new intervention, making this a patient-centred measure, (ii) 10MWT has been used routinely to monitor patient mobility in all four HTLV treatment centres and (iii) 10MWT is a validated disability outcome measure used in other trials of patients with impaired mobility [6], such as patients with stroke [7], spinal cord injury [8], Parkinson’s disease [9] and multiple sclerosis [10].

Healthy individuals are reported to complete the 10MWT in a mean of 6.84 s [11]. In a study of HAM/TSP patients, the overall mean 10MWT in 29/49 (81 %) patients increased by +4.25 s/10 m in 12 months (range −0.13 to 24.6, median 1.27). 10MWT deteriorated even in patients who walked without any need for further aid by a median of +1.98 s/10 m/year^3^. It is worth noting that the measurement of 10MWT varies considerably within and between patients on a daily basis, over time, and is operator-dependent.

The standard method to calculate the sample size of a clinical trial is the power calculation [12]. In order to be able to calculate the sample size required to have a high probability to refute the null hypothesis, should the treatment really be effective compared to the comparator, the inter- and intra-patient variabilities of 10MWT over time needed to be taken into account. To this aim, the HAM/TSP CTSG submitted prospectively collected 10MWT data of HAM/TSP patients to two biostatisticians.

## Methods

10MWT data collected as part of HAM/TSP patients’ clinical routine workup at four HTLV treatment centres were submitted for use in the sample size calculation. Anonymised data were submitted from the HTLV Centre at Bahiana Medical School, Salvador, Brazil; Department of Rare Diseases Research, Institute of Medical Science, St. Marianna University School of Medicine, Japan; National Centre for Human Retrovirology, St. Mary’s Hospital, Imperial College Healthcare NHS Trust, UK; and Neuroimmunology Branch, NIH, USA. In communication with local research and/or ethics review committees of all four countries, it was agreed that patient consent and committee permissions were not required. The UK’s Health Research Authority guidelines advise that the secondary use of data collected for clinical purposes can be used in research without requiring ethical review, provided the data are anonymised to the research team [13].

Included were 10MWT data on patients with definite HAM/TSP as defined by Belem Criteria [4], >18 years old, who could walk 10 m, had at least two 10MWT measurements 6 months apart. Data on gender, age, duration of HAM/TSP and walking aid were collected.

10MWT was measured by trained clinical staff following internationally used standard operating procedures, by measuring the time a patient needed to walk a straight 10-m long line in seconds [3, 14].

### Statistical considerations

For use as an outcome variable in a trial, we need to describe and to check the distribution of 10MWT. If the distribution is non-normal, a transformation such as the logarithm or square root function can be applied to get a normal distribution. Data would be suitable for statistical analysis if the scatter plot of 10MWT at 6 months against 10MWT at baseline gives a uniform cloud of points. The correlation between baseline and 6-month measurement was calculated (*r*). For sample size calculations, the standard deviation (SD) at 6 months was multiplied by √(1 − *r*^2^) to allow for the effect of analysis of covariance on the baseline measure [15].

## Results and discussion

Longitudinally collected 10MWT of patients with definite HAM/TSP, spanning 6 months, were included. Matched 10MWT at baseline and at 6 months were available for a total of 76 patients: three subjects from Brazil, 41 from Japan, 27 from UK and five from the USA.

Demographic data were available for 70 (92 %) patients: 59 (84 %) were female, mean age was 54 years (range 20–83), mean duration of HAM/TSP was 11 years (1–37), 22 (31 %) patients did not need a walking aid, 25 (36 %) needed one walking stick, 16 (23 %) needed two and 2 (3 %) a tripod.

At baseline, the mean and SD of 10MWT was 23.5 (18.9) s/10 m and the median 16.3 s/10 m. At 6 months, the mean (SD) 10MWT was 24.9 (23.9) and the median 16.4 s/10 m.

**Fig. 1:**
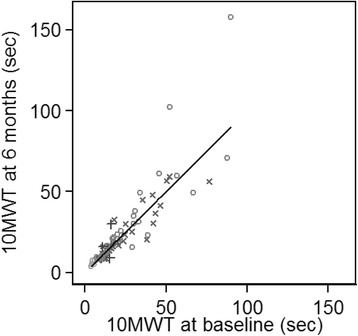
Measured 10MWT at baseline and 6 months, with line of equality. *Different shapes* represent patients from different countries. 10MWT = 10-min walking time; sec = seconds

**Fig. 2:**
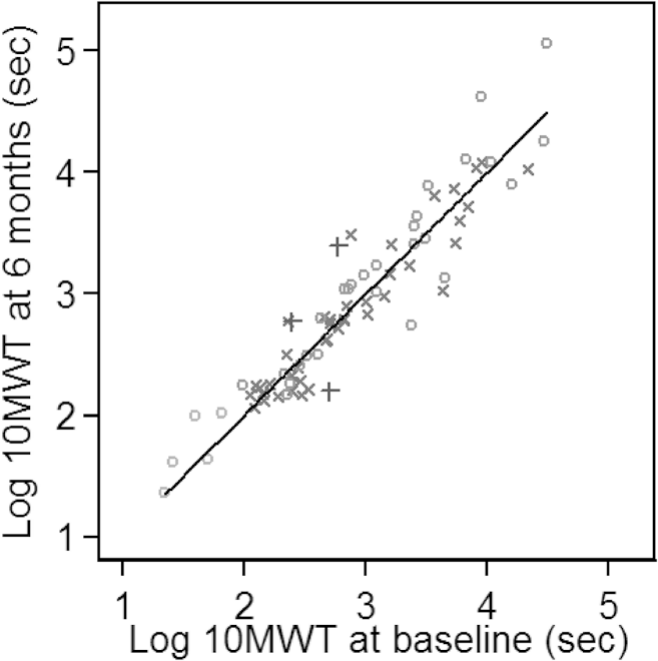
Log_e_10MWT at baseline and 6 months, with line of equality. *Different shapes* represent patients from different countries. 10MWT = 10 min-walking time; sec = seconds

Figure 1 shows a scatter plot of 10MWT at 6 months against the baseline measure. Clearly, the variability increases with increasing value of the measurement, and both variables show a positively skewed distribution. Figure 2 shows log transformed data, where far more uniform variability is seen.

The untransformed data showed strong correlation (*r* = 0.875, Fig. 1); however, this improved by using a log scale instead, where a linear correlation between baseline and 6-month 10MWT was observed (*r* = 0.938, Fig. 2). The baseline mean (SD) and median log_e_ 10MWT were 2.88 (0.720) and 2.77 and at 6 months 2.90 (0.743) and 2.79. It was estimated that the SD of log_e_10MWT after adjustment for the baseline measurement was 0.258. The mean (SD) % deterioration of 10MWT over 6 months was an increase in time of 5.74 % (28.2 %).

**Fig. 3:**
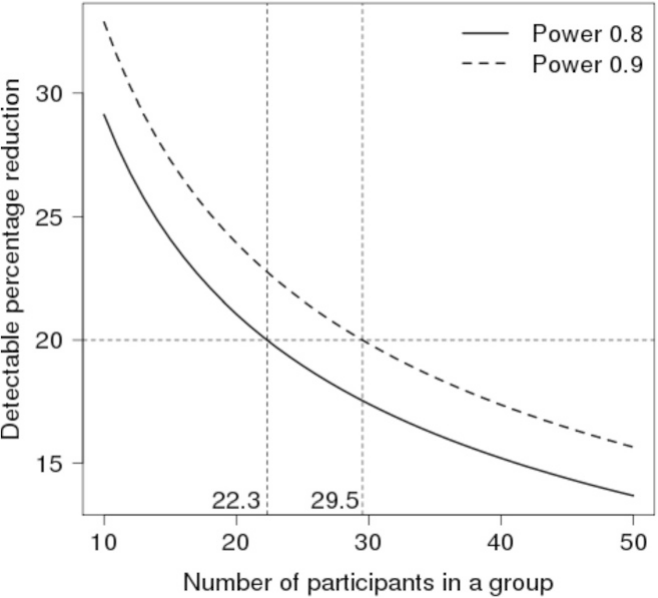
Detectable change (decrease) against sample size for two chosen powers

Table 1 shows the detectable change in 10MWT calculations, given a fixed sample size, for the 10MWT in patients with HAM/TSP for SD of ±0.2, 0.26 and 0.3, using log transformed data. With 30 participants per group and SD = 0.26, a power of 90 % to detect a decrease due to an intervention in 10MWT of 19.85 % at 6 months can be achieved. To power the trial at 90 %, a minimum of 33–35 patients are needed in each arm, to allow for 10–15 % estimated trial drop-out rate (Fig. 3).

**Table 1:** Detectable change calculations given fixed sample size for the 10MWT at 24 weeks

	Number of participants in one arm	Detectable change (%)	
SD		Power 0.8	Power 0.9
0.2	20	16.62	18.97
	25	14.94	17.07
	30	13.68	15.65
	35	12.70	14.55
	40	11.91	13.65
	45	11.26	12.91
	50	10.70	12.27
0.26	20	21.05	23.93
	25	18.96	21.60
	30	17.41	19.85
	35	16.19	18.48
	40	15.20	17.37
	45	14.38	16.44
	50	13.68	15.65
0.3	20	23.87	27.06
	25	21.54	24.48
	30	19.80	22.54
	35	18.44	21.01
	40	17.33	19.76
	45	16.40	18.72
	50	15.61	17.83

### Discussion

A double-blind randomised controlled trial (DB RCT) gives the highest level of evidence and is particularly important when testing novel treatments in conditions that vary, progress slowly or are subject to placebo effect. Although endemic in parts of the world, globally HAM/TSP is a rare disease, which makes large DB RCTs difficult to conduct [5]. This is one of the reasons why although there are estimated 300,000–450,000 HAM/TSP patients globally, there is no internationally agreed specific treatment. Since HTLV was discovered and associated to HAM/TSP, there have been only two RCTs [16, 17] of which only one was double-blind [17].

In order to provide HAM/TSP patients with a DB RCT, we developed several strategies. An international consortium was established, pooling expertise from HTLV centres based on four continents, to prioritise and sequentially test potential interventions for HAM/TSP. 10MWT was proposed as a clinical outcome measure which would be meaningful, reliable and easy to collect at different trial sites. Data obtained from patients under follow-up at each centre were collated to allow robust power calculations to ensure optimal study design. These longitudinal data on 10MWT were used to calculate the inter- and intra-patient variability.

Given the rarity of the condition, power calculations should be based on robust data to ensure efficient utilisation of scarce resources, human and financial. Data are presented on the predicted size of effect against required sample size. The selected sample size of 33 patients per arm for the planned comparison of active treatment with placebo has 90 % power to detect a 20 % difference in time taken to walk 10 m, adjusted for baseline.

10MWT had a positively skewed distribution, and the logarithmic transformation was effective in producing an approximately normal distribution. As a result, differences are presented as logarithms and back transformation produces ratios. This is why detectable differences are presented as percentages rather than differences in seconds.

This study’s strengths are that data were collected longitudinally, including patients from all four continents, thus sampling the target HAM/TSP patient group. In addition, the sample size is fairly large for a disease this rare. 10MWT may vary with gender, age, walking aid and duration of disease. However, these variables are expected to affect the baseline and the outcome variable 10MWT in the same way. Hence, adjustment for baseline 10MWT removes the need to adjust for other variables.

Although there is bound to be an operator-dependent variability of 10MWT, if the same operator carries out the standardised baseline and outcome measurements in the same patient, this will automatically be adjusted for by the design.

## Conclusions

In conclusion, this is the first study to report fixed sample sizes calculated for three different SDs of log_e_10MWT for clinical trials for patients with HAM/TSP powered at 80 or 90 %.
